# Diagnosis and Treatment Based on Functional Movement Impairment: A New Model of Functional Restoration Directed to Correction of Pathoanatomic and Pathophysiology of Tissues-Part I

**DOI:** 10.31661/gmj.v13i.3094

**Published:** 2024-03-01

**Authors:** Majid Shahbazi, Hossein Rafsanjani DehQazi

**Affiliations:** ^1^ Department of Physical Therapy, School of Paramedical and Rehabilitation Sciences, Mashhad University of Medical Sciences, Mashhad, Iran

**Keywords:** Musculoskeletal Disorders, Classification, Pathophysiology, athoanatomic, Functional Movement Impairment, Tissue Disorder, Model, Physical Therapy

## Abstract

One of the most prevalent diseases in humans is musculoskeletal dysfunction. The source of these problems is usually multifactorial, including mechanical, psychological, and chemical. A specific diagnosis is rarely achieved for most musculoskeletal disorders, especially chronic conditions. This can be one of the reasons for the ineffectiveness of the various treatments offered. Various models have been proposed to address this problem to make more accurate diagnoses and effective treatments. Each of these models has its limitations. It seems that the previous models had limited information, such as the type of damaged tissue, the stage of tissue repair, the functional aspect of the disorder, other parts of the movement chain disorder, psychological features, and a lack of correlation between medical diagnosis and physiotherapy diagnosis. In the present study, an attempt is made to provide a new and more comprehensive model by considering the limitations of other models. In the first step, the therapist or physician identifies the biomechanical source of the disorder. The patient’s functional movement impairments are identified, and a Neuro-Musculo-Skeletal disorder related to functional movement impairment is assessed. Finally, to label the patient’s problem, medical (Patho-Anatomical Model) and physical therapy (Movement System Classification Models) views are considered. Therefore, the medical diagnosis of the patient is expressed along with the joints that play a role in aggravating the patient’s symptoms with movement. One case study is provided at the end to clarify the method of diagnosis. Future research is necessary to improve the validity of the methodology.

## Introduction

**Figure-1:**
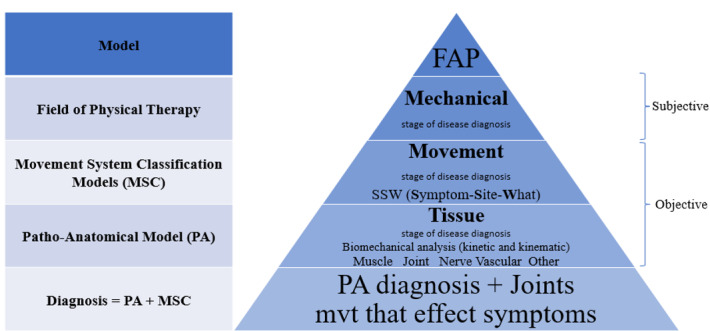
Figure 1. The process of “diagnosis” is used to establish the relationship between functional movement impairment and tissue impairment (anatomic and physiologic) in the function to anatomy and physiology (FAP) model

**Figure-2:**
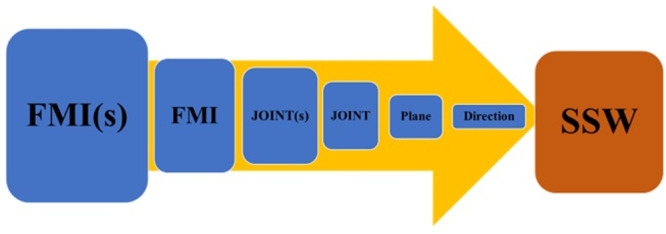
Figure 2. SSW (symptom, site, and what) determination process

**Figure-3:**
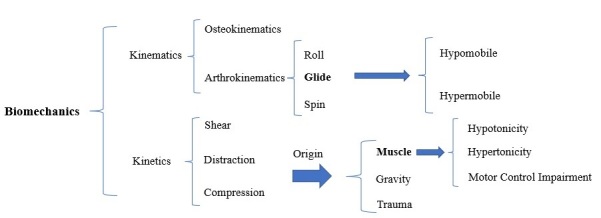
Figure 3. Biomechanical analysis of movement (kinetically and kinematically)

The study and treatment of human movement disorders are one of the foundations of the physical therapy profession [1][2]. For normal human movement, multiple organs’ activities and the effective interaction of organs are imperative. These organs include the musculoskeletal, gastrointestinal, cardiorespiratory, cardiovascular, genitourinary, nervous, and metabolic systems [3]. Organs’ activity and their interaction are affected by various etiological complexes. They include various physical, psychological, and biochemical factors. The musculoskeletal system is directly responsible for creating movement [3]. Musculoskeletal diseases (MSD) are the main cause of functional movement impairment (FMI). A significant contributor to morbidity, disability, and financial loss is musculoskeletal pain [4]. To manage MSD, "the right treatment to the right patient at the right time" should be performed [5].

Therefore, a correct diagnosis is a prerequisite to using the best available treatments and predicting patient outcomes [5]. The final purpose for processing must be to restore the FMI. The accuracy of the diagnosis is frequently challenged, even when made with specificity. This challenge leaves a gap in diagnosis and management [6]. It is difficult to treat pain and disability without clear, active primary underlying pathology [7]. The results of published studies of the most widely used treatments are extremely similar. Compared to no intervention or a sham procedure, most interventions for example Cyclooxygenase-2 (COX2) inhibitors, educational programs for managing back pain, gradual relaxation techniques, physical exercise-based therapies, and comprehensive, multidisciplinary treatment approaches produce modest, short-term advantages [8][9]. Unsatisfactory clinical trial outcomes can be interpreted in one of two ways. First, there is a potential that the clinical trials are inaccurate. That is, the clinical trial method undervalues the genuine effectiveness of the current treatment and fails to accurately reflect the reality of the clinical situation for various reasons [8]. One reason for the mentioned causes is the heterogeneity of the patients.

As has been shown for chronic low back pain (CLBP), categorizing chronic conditions into homogenous groups and using specialized therapies tailored to these groups may improve treatment efficacy [6][7][10][11]. Many medical professionals contest the findings of clinical studies because they believe the lack of efficacy conflicts with how they have previously treated patients with back pain. The apparent variety of patients with persistent non-specific low back pain (NSLBP) is a common explanation for this difference [8]. Due to potential disparities between subgroups, previous studies that considered all NSLBP patients as a single, homogenous group run the danger of having their data "washed out" [10][12][13]. To improve patient outcomes, it has been a significant objective over the past few years to classify persons with low back pain (LBP) into homogeneous populations or "subgroups" with shared characteristics [14]. Subgrouping could further improve communication and reduce ineffective treatment variations [15]. It is generally recognized that patients who receive care appropriate for their sub-classification category find more success than those who do not [10].

Various models have been proposed to homogenize patients. Many of them focus on a specific aspect of the disease [6]. The pathoanatomical (physician model) and movement-based models are the most common of these models [5]. Secondly, the clinical trials are right, but the existing methods for managing chronic low back pain are ineffectual; in other words, treatment has failed because it was misdirected [8][16]. Therefore, the broad classification of NSLBP must be broken down into subgroups. Since the classification based on anatomical and physiological abnormalities did not work, attempts have been made to find subgroups based on symptoms and physical signs [11]. Due to the limitations of the current models specially pathoanatomical model [5], physiotherapists tend to use movement-based classification models to treat their patients [2][5]. Therefore, this difference of opinion between physiotherapists and physicians may lead to decreased communication between these groups and ultimately reduced patient benefits [5]. Thus, in this study, the author proposes an alternative and an umbrella model, first by understanding movement-based classification based on the FMI and second by connecting the FMI and the pathoanatomical model with a focus on Mus­culoskeletal diseases (MDS). This model may open up the possibility of different approaches to issue-solving. Other models and their shortcomings in identifying plausible underlying processes for FMI diseases are described below. As a result, identifying the mechanisms involved is especially important [7].

1. The Models and Their Limitations

Diagnosis is a procedure that is not the sole purview of any profession. Every time a physical therapist evaluates a patient, findings are grouped, data is interpreted, and patient concerns are identified [17]. A physical therapy diagnostic procedure is still being developed. Due to the need for a precise diagnostic system, numerous studies have produced contradictory results about the therapeutic efficacy of patients [18]. Different models of patient evaluation in physiotherapy have been presented to reach a proper diagnosis [6][19]. Despite the different models published, there are some drawbacks to diagnosing and treating patients referred for physiotherapy. The diagnosis and treatment models employed for patients, such as the Peripheral Pain Generator, Psychosocial, and Mechanical Loading models, exhibit certain limitations. Firstly, the Peripheral Pain Generator model addresses pain symptoms without delving into the underlying mechanistic factors. Secondly, the Psychosocial model pertains to a restricted subgroup of cases where these elements assume a predominant or primary pathological role in the disorder. Thirdly, the Mechanical Loading model predominantly focuses on ergonomic and environmental factors. For more details, refer to the article by Peter O’Sullivan [6]. Given the importance of pathoanatomical and movement system classification model, these two classification systems are further explained in the following:

1.1. The Patho-anatomical Model and its Limitations

The most common diagnostic for musculoskeletal issues identifies a specific tissue disease as the basis or genesis of the patient’s pain or dysfunction [5]. The pathoanatomical model is based on a traditional medical approach, and its purpose is to determine the tissue damage or abnormal physiological processes responsible for the disease [6]. In this approach, patients are classified according to the presumed structure that is injured or painful. Imaging techniques are used to help identify the structure that has been compromised [20]. Medical images (such as MRI or X-ray) or surgical verification are the gold standards for confirming certain diseases [5]. Radiological findings often lack correlation with pathoanatomical findings and clinical symptoms [21]. The high expense of diagnostic imaging is one of the most important concerns with pathoanatomic models [5]. The pathoanatomical model frequently focuses on the symptomatic tissue(s) [7]. This approach, which concentrates on the impact of loads on structures rather than their causes, needs to be revised for early identification or, preferably, disease prevention [5]. Treatment that only addresses the symptomatic structure does not consider the disorder’s multifaceted character or all of the underlying processes [7]. Also, there are other obstacles within this pathoanatomic diagnostic paradigm. Numerous pathoanatomical symptoms coexist, assuming the clinician arrives at a correct pathoanatomic diagnosis; these diagnostic labels have minimal potential to guide the selection of therapies [22]. Pathoanatomical mechanisms are less critical in CLBP than in acute ones [23]. Notwithstanding such shortcomings, one of the most prevalent reasons for preserving the pathoanatomic model as the diagnostic paradigm is that physicians currently use it. It has been argued that introducing an unfamiliar diagnostic framework could impede communication with physicians and other health professionals [5].

1.2. Movement System Classification Model and its Limitations

Movement is acknowledged as a fundamental or crucial topic in physical therapy [24][25] that could be established as both a theoretical and practical idea that can explain the particular fundamentals of physical therapy (PT) [2]. In complex PT practice, the relation between theoretical growth and the comprehension of body movements reflects the notion of evolving movement as a fundamental concept. Movement is discussed concerning functional ability, health, quality of life, and the interactions between motion and environmental, social, psychological, and physical aspects [26]. Despite the broad perspective of PT in practice, PT needs conceptual models, which are required to tightly link research, teaching, and practice based on a comprehensive conception of human, health, and welfare [27]. Movement and function are the two main ideas that the World Confederation for Physical Therapy (WCPT) uses to define physical therapy. Physical therapy offers services to people of all ages to improve, preserve, and regain their full range of motion and functional capacity. What it means to be healthy is fundamentally based on functional movement [28]. Classification schemes that concentrate on directing particular treatments have emerged within the field of physical therapy, a profession with an extensive understanding of neuromusculoskeletal evaluation. Most classification schemes examine the link between movement and pain [2].

Despite evidence that patient treatment based on subgrouping produces better results than treatment using clinical guidelines [18][29], studies of physiotherapists’ practices have found a low utilization of classification systems. There is much variation in how LBP presents itself. However, if similar characteristics show up in the evaluation that serves to separate one discomfort profile from the other, they may help in initial decision-making by identifying a dysfunction pattern that is targeted for intervention [30]. Different classification techniques have been used by various disciplines to try and separate LBP subgroups [14].

Diverse viewpoints are taken, with an emphasis on enhancing interprofessional communication, an investigation of the musculoskeletal or neurological system, an evaluation of psychosocial issues, or efforts to integrate assessments of numerous systems to varying degrees [14]. Typically, physical therapists evaluate and treat patients for movement-related impairments instead of specific tissue abnormalities [5][31]. Movement-based classifications use movement as their central focus to divide musculoskeletal disorders into uniform categories [32]. These models usually didn’t consider treatment at the cellular level, all stages of tissue restoration, all defined physiotherapy services for treating patients’ disorders, and external stimulating elements and a lack of connection between medical diagnosis and physical therapy diagnosis are some of them [5][6]. Physical therapists noted a lack of usage despite the benefits of using movement-based classification systems in diagnosing and treating LBP patients [18]. This could be explained by a need for more expertise with these methods, a challenge in selecting acceptable classification schemes directly related to a particular patient condition, or a preference for using another assessment method [14][18]. Suggested models are not comprehensive for other musculoskeletal disorders and focus more on the LBP [18]. There is a lack of agreement regarding using movement-based classification methods in diagnosing individuals with LBP [18]. These classification schemes include the O’Sullivan classification scheme (OCS), mechanical diagnostic and treatment (MDT), and movement system impairment (MSI) [18][33]. According to the subgroups and training level, inter-tester reliability for different schemes ranged from "poor to fair" (PBC), "moderate" (MDT, Treatment-based classification (TBC), OCS), "substantial" (MSI), to "excellent" (OCS) [14].

3. Function to Anatomy and Physiology

According to the initial portion of the discourse, an alternative model for comprehending MSD will be offered due to the limitations of the current theories [6]. This attempt is made by considering possible weaknesses in the existing approaches. Researchers and clinicians may have to fundamentally reevaluate the type of problem and the best approach to solving it. It attempts to persuade clinicians to think about alternate methods for diagnosing and managing MSD. The current theory supports the significance of biological, emotional, and environmental components in the etiology, aggravation, and continuation of chronic pain [34]. Thus, this practical model integrates clinical patterns based on functional movement impairment (FMI) and analyzes and manages them with related pathoanatomy and pathophysiology. In this method, the intervention is performed at the tissue and cell level, but the evaluation is performed at the functional level. Several steps in this model are suggested for assessing and managing these patients, but in this article, the focus is on the diagnosis process.

4. Diagnosis Process

To improve the accuracy of the classification system, "diagnosis" is utilized to establish the association between tissue impairment (anatomic and physiologic) and functional movement impairment [35]. However, these clinical evaluations have yet to be investigated formally. It involves subjective and objective evaluation of the condition [36]. In the first step of this four-step procedure (Figure-1), it must be determined whether the patient falls within the scope of physiotherapy. It must have a mechanical origin, or the mechanical origin must be predominating. Tissue dysfunction can have a mechanical or no mechanical origin, or both, with one predominating [6]. No mechanical causes of neuromusculoskeletal disorders (NMSD) can be separated into two categories: psychological, such as movement phobia, anxiety, and depression, and non-psychological [37], such as inflammatory, metabolic, micronutrient, allergy, environmental, infectious, genetic, neoplastic, vascular, and rheumatologists’ remaining many systemic disorders [37][38] and brain tumors [39].

The relation of these factors with movement disorders can be explained with the pathokinesiology model [25]. In the second step, the direction of the movement disorder, the type, and the location of the symptom is determined. The involved anatomy(s) is determined in the third phase according to the selected movement disorder. The neuromusculoskeletal system comprises articulating bones, cartilage, ligaments, the capsule (the passive subsystem), muscles that regulate movement (the active subsystem), and nerves that control movement (the neural control subsystem) [40]. Each component of these systems must function optimally for precise, regulated movement [41]. It is suggested that the source of functional movement impairments in patients is NMSD. According to the Panjabi model, the disorder of each element of this system can be compensated by another element [40]. The NMSDs are separated into primary and secondary types. In the primary group, the disorder directly affects the neuromusculoskeletal (NMS) system, whereas, in the second group, the musculoskeletal system is impacted by disorder in other body systems. Consequently, in the course of treatment, in addition to the primary injured tissue, the compensation of the other tissues should be treated if doing so improves function.

5. Mechanical Stage of Disease Diagnosis

In this stage, the therapist pursues several goals while obtaining information through subjective findings. The most important of them include the determination of the primary source of the disease (mechanical or non-mechanical), the essential patient’s FMI(s), the stage of tissue repair, the type of trauma, and at least one external factor [42][43]. For these purposes, the clinician should take note of the patient complaints that show the mechanical origin of impairment, such as the function(s) that cause, aggravate, or ease the symptom(s). What is the mechanism of the problem? Did trauma cause the onset, and is there anything that relieves the symptoms? [44] Is discomfort related to resting? Activity? A mechanical issue is typically indicated by activity-related pain that subsides with rest. What is the type of force that aggravates symptoms? [36]. It is reported that most causes of MSD have a mechanical origin; for example, 98% of LBP may be caused by mechanical factors, and the other 2% are caused by non-mechanical factors [45].

Mechanical MSD patients are subdivided into chronic repetitive microtrauma and acute macrotrauma mechanisms [46][47]. Macrotrauma can be subdivided into two types: a. direct trauma; b. indirect trauma [48]. A direct trauma injury is the term used when damage happens at the point of impact, like a forceful kick to the shin [49]. When damage occurs in a place other than the point of impact, it is said to result from indirect trauma avulsion fracture. Microtrauma can be due to specific activities that are overused, misused, abused, or disused [50][51][52]. The author believes that at least one external factor, such as the patient’s ergonomic conditions, is required to initiate or continue tissue damage, so it is imperative to prevent the recurrence of symptoms; the therapist identifies the external factor [53]. Finally, the evaluation and treatment are started if the patient has a functional disorder with mechanical dominance. The patient with non-mechanical disorders is referred to other specialties related to the person’s disease. There is mounting evidence that non-physical factors, including psychological [36][54][55][56][57], biochemical [58][59][60][61], and cold [35] factors, contribute to chronic musculoskeletal pain. Eventually, the therapist chooses an FMI from the patient’s most functional limitations based on various factors to continue diagnosing.

6. Movement Stage of Disease Diagnosis

This stage of the diagnosis process is consistent with movement system classification model. Its goal is to determine the movement that should be biomechanically analyzed and is a baseline for assessment and reassessment (Figure-1). Since each movement has its biomechanics [62], the disorders of each movement have their characteristics, so determining the disordered movement for knowing the tissues involved and providing appropriate treatment is essential. Thus, PT should conduct a more in-depth observation and study of patient mobility during a functional task [63]. Promoting optimal human function and health is the main objective of the healthcare profession of physical therapy [64].

7. Selection of A Functional Movement Impairment

The patients may have different FMIs as a result of disorders. In the first stage, the therapist should select an FMI among the FMIs that the patient reports. For selecting an FMI, the therapist should consider some parameters. The most crucial factors in choosing an FMI to begin analysis and treatment are that the patient’s symptoms worsen instantly after the movement, and its correction plays a significant role in the patient’s independence. Restoring function is crucial to the patient’s quality of life, and the patient, with various functional limitations, first reports that limitation. In the following, the clinician or therapist determines the joint that significantly affects the change of the patient’s symptoms from among the weight-bearing and movable joints that affect the change of the patient’s symptoms in the selected function [42]. Then, the movement plane and direction that most affect the symptoms are determined in the selected joint. Finally, the SSW is identified. The SSW is the abbreviation of Symptom, Site, and What, which indicates the type of symptom and its location based on the selected movement direction (Figure-2). Instead of selecting a functional movement impairment, the therapist selects active or passive physiological movement, or palpation, for biomechanical analysis based on the patient’s condition, such as the severity of the disease and the patient’s inability to interact with the therapist.

8. Tissue Stage of Disease Diagnosis

8.1. Definition of the Involved Anatomy and Physiology

The affected anatomy is identified subjectively and objectively based on the direction of movement. It must be remembered that only a tiny percentage of MSD pain can receive a specific diagnosis [46][47]. The primary effectors of movement are the skeletal, nervous, and muscular systems, which are crucial in movement [63]. Therefore, physiotherapists should focus on dysfunctional anatomy (muscles, joints, and nerves) associated with the movement selected for the diagnosis and treatment process.

8.2. Subjectively (Patient Feels)

One of the most crucial ways to diagnose the involved anatomy is to conduct a biomechanical analysis of the SSW that causes and exacerbates the patient’s symptom(s) (Figure-3). The mechanism of the injury, the kind of force that causes or aggravates symptoms, the location and behavior of the symptoms, observation, and other techniques can all be used to assist in the anatomy diagnosis. Additionally, other factors, including the patient’s work, age, and gender, can be somewhat helpful in determining the implicated anatomy [36].

8.3. Objectively (Examiner Findings)

The subjectively determined suspected diagnosis about the implicated anatomy (muscle, joint, and nerve) is utilized to support or contradict the objective evidence. Since the nervous, muscular, and skeletal systems are the main components of movement, the assessment and treatment of anatomical and physiological disorders of these tissues play a primary role in correcting movement disorders. The Change of Alignment (COA) is used to gather information and determine the involved anatomy that affects the symptoms: these cover the anterior-posterior, internal-external, and upper and lower directions in six primary and combined directions. They can be performed on the joints or the soft tissue. The change of direction in the joints is known as the Mulligan technique [65]. However, by altering the direction of the soft tissue, the therapist may also explore the movement disorder’s interpretation and anatomy. For further confirmation, the therapist uses specific tests. Also, the therapist can confirm or reject their initial diagnosis based on the results obtained from the treatment of the involved tissue. As stated at the beginning, the neuromuscular-skeletal system is directly responsible for creating movement [3]. Therefore, anatomical diagnosis related to movement focuses more on these tissues, especially muscles and muscle chains

8.4. Muscle and Muscle Chain

There are about 600 muscles in the human body [66]. Locomotion is one of the essential functions of skeletal muscle [67]. Muscle activity patterns vary significantly between movements, and the activation strategies alter according to the specifics of the movement [66]. It is well known that muscle activity patterns affect movement direction [68]. In practically every movement, multiple muscles are required to produce the motion [69]. Muscular chains are muscles that impact or interact with one another through movement patterns [70]. The muscles that work together as a functional group fall into four major categories [71], including agonist, synergist [69][70][72][73], antagonist, and fixator [69][72]. As mentioned above, the dysfunction of each part of the NMS system can be compensated with other parts, as described in the Panjabi model [40]. According to some data, arthritic damage may develop first, followed by muscle weakness [41]. Several studies demonstrate that intervention on skeletal muscles has a good effect in various conditions. For example, exercise regimens appear safe and effective in individuals with knee osteoarthritis, especially regarding pain and strength improvement [74][75][76]. It is also known that the hamstring muscles play a significant role in making up for the instability loss in an anterior cruciate ligament (ACL)-deficient knee [77]. Muscle disorders can be caused by muscle involvement alone or by the involvement of other tissues. So, based on this, the author divides muscle disorders into primary and secondary.

Joint and Joint Chain

The joints are one of the most critical tissues directly responsible for movement. It was shown that disorders of joints profoundly affect movements. The articular chains throughout the skeletal system keep the skeleton stable while moving [70]. The biomechanical interactions between various joints produce articular chains during movement [70]. In order to conduct a joint movement, they often play either a static or dynamic role. The location and posture of the joints influence the performance of these two functions. The activity of the muscles is affected by the location and motion of the joints, and conversely. Frequently, the disease is associated with compensating malfunction in the kinetic chain [70]. Kibler understood that every modification to timing or force generating could lead to subpar performance or disease at a lower level in the chain [78]. There are several structures in the joints that, if disturbed, can affect the function of the joints and, as a result, the muscles associated with them. It has been demonstrated that patients with LBP typically have restricted or changed hip range of motion. These patients usually experience an improvement following surgical treatment for hip pathology [79][80]. The research revealed a considerable increase in lateral scapular rotation in patients with shoulder pain and limited range of motion. It is typically considered a compensatory strategy for reduced glenohumeral motion [81]. Eventually, the examiner should determine the joints contributing to FMI and the plane and direction of that joint that modify the patient symptoms [42].

8.5. Neural Chain

In this model, the nerves that supply the muscles responsible for movement are recommended to be released in the relevant segment at the spinal cord level and in critical zones. Several studies show that manipulation of the neck and thoracic region affects parameters such as nerve conduction velocity and electromyographic muscle activity [82]. Thoracic manipulations and methods like craniosacral treatment, which impact the sympathetic and parasympathetic nervous systems, may help some people with neuropathic pain experience a reduction in their symptoms [83][84][85]. The author believes that in neuropathic pain, usually, no specific movement changes the patient’s symptoms.

8.6. Other Systems

Other systems, such as the vascular and visceral systems, can play a role in causing movement symptoms in patients, and manipulating these systems can help improve movement [33].

9. Diagnosis

In this model, to label the patient’s problem, both medical and physical therapy views are considered. As a result, the patient’s medical diagnosis is expressed with the dynamic and weight-bearing joints that alter the patient’s symptoms. For example, in a patient with the diagnosis of tennis elbow, the movement of the shoulder, elbow, and wrist joints aggravates the symptoms. In another patient with the same medical diagnosis, only the wrist movement played a role in aggravating the symptoms. In the first patient, the diagnosis is written like this: tennis elbow (W(wrist)-E(elbow)-S(shoulder)); in the second patient, the diagnosis is written like this: tennis elbow (W(wrist)). Abbreviations in the diagnosis indicate the joints causing the symptoms. In conditions where the movement of several joints contributes to the aggravation of the symptoms, the joint that has the most significant effect on the change of symptoms is first mentioned. Then, the joints that have a lesser effect are listed in order.

## Conclusion

This novel method establishes a diagnosis based on pathoanatomical and pathophysiological elements related to functional movement impairment. It has four primary stages for diagnosis, including mechanical, movement, tissue, and labeling. In the mechanical stage, the main goal is to determine the primary origin of the FMI, which can be mechanical, psychological, or biochemical. In the movement stage, consistent with the MSC, the goal is to determine an SSW to perform a biomechanical analysis to define the anatomies involved. The tissue stage, compatible with the pathoanatomical model, begins with a biomechanical analysis of SSW. Then, muscles, joints, and nerves related to SSW are defined. In the labeling stage, to label the patient’s problem, both medical and physical therapy views are considered. As a result, the patient’s medical diagnosis is expressed with the dynamic and weight-bearing joints that change the patient’s symptoms. In this method, functional movement, the highest level of movement [42], is selected as the evaluation criterion; in other words, the treatment is performed at the tissue and cell level, but the result of the intervention is checked on the functional movement. This model attempts to connect the physician’s (pathoanatomical) and the physiotherapist’s (movement-based) models. It requires more investigation and encourages primary research to support the prevalent clinical trends.

## Case Report

A 32-year-old female patient was referred to physiotherapy with a patellofemoral pain syndrome (PFPS) diagnosis. The first stage (mechanical): The patient complained of knee pain lasting approximately three months. The patient speculated that the development of her knee pain was related to her workplace changing and increasing the use of stairs. She also said that she experienced worsening knee pain on workdays when she must climb and descend stairs. During the visit, the patient complained about long-distance driving, using the traditional toilet (squatting position), and walking up ramps. The patient had a regular medical history, and most of the symptoms associated with the yellow flag were absent. There was nothing remarkable about the knee’s appearance. The mechanical cause of the condition, various functional movement impairments, the microtrauma mechanism, the subacute tissue repair stage, and a potential external factor aggravating the symptoms of stair use at work were found in this stage. In the second stage, squatting was selected as a functional movement impairment that aggravated her symptoms.

The patient was asked to squat. At a knee flexion angle of roughly 40 to 50 degrees during the squat, her problems became worse. The squat movement was then re-evaluated, and the change in symptoms was verified by rotating the hip joint either internally or externally and by positioning the patient’s foot in supination or pronation. It was found that the patient’s symptoms were made worse in this case by the hip’s internal rotation and foot pronation. Most symptom changes were related to internal hip rotation, knee bending, and ankle pronation, respectively. Findings at this stage: In this patient, the sagittal plane movement of the knee in the direction of flexion while in the weight-bearing posture, which results in pain in the front of the knee, was suggestive SSW. Third stage: Consistent with the pathoanatomical model, the involved anatomies were identified based on the chosen movement in the previous step. Biomechanical analysis of the chosen movement was carried out to ascertain the kinetics and kinematics and the anatomies involved. For this purpose, the tibiofemoral, patellofemoral, and tibiofibular joints’ arthrokinematics were assessed in the first stage in the usual arthrokinematics direction of the joint during the movement of knee flexion along with the patient’s symptoms change. The present patient’s posterior tibia, medial patella, and anterior fibula glides reduced her symptoms. Then, the effect of the change of alignment of muscles related to selected movement on symptoms was assessed. The COA of lateral hamstring muscles, tensor fascia lata, and rectus femoris were influential in improving the patient’s symptoms. The selection of involved tissues and the direction of their involvement were determined according to the selected functional movement impairment.

The fourth stage of the FAP model was the diagnosis: the diagnosis was PFPS (K(knee), H(hip), A(ankle)), PFPS indicated a medical diagnosis, and (K, H, A) showed the effect of the movements of the knee, hip, and ankle joints in changing symptoms, and the knee, hip, and ankle, respectively, had a more significant role in changing symptoms.

## Conflict of Interest

The author declared that he has no conflict of interest.
